# Medical Opinion and Movement

**Published:** 1906-09-22

**Authors:** 


### Medical Opinion and Movement.

Of 34,600 cases of primary scarlet fever treated in the Metropolitan Asylums Boards hospitals Dr. Turner reports the percentage of outbreaks following the discharge of a case to be 3.27.

It is estimated on authority that 40,000 infant children lost their lives in the large towns in 1903 from preventable causes, due mainly to slums in cities. The National Housing Reform Council are urging the electors to organise themselves to secure the election of those candidates only at the municipal contests now approaching who pledge themselves to a programme of slum destruction and slum prevention.

The *Times* has inaugurated a discussion on the gospel of recreation. It is maintained with great force and truth that by comparison the majority of holidays taken by every class of society are mainly wasted. Mr. Jonathan Hutchinson rightly contends that to abstain from all labour by way of holiday is merely a negative and formless plan, and that no one can take a real holiday without a scheme of active duties. He is in favour of organising intelligent occupation and entertainment for summer visitors at all centres of attraction. His idea is to set up educational museums on a large scale, made instructive and interesting by the exercise of brains and a system whereby frequent changes of specimens, models, portraits, and exhibits generally may be maintained.

We hope that the Home Secretary will speedily summon a conference between the Home Office, the London County Council, the Bischoffsheim Street Ambulance Association, and the police, with the view to the establishment of an adequate system of ambulances through the whole of the metropolis. It will be remembered that the House of Lords rejected the clauses inserted by the London County Council in their Omnibus Bill last session to enable them to experimentally introduce street ambulances into London. The rejection of these clauses was attributed mainly to Governmental action, and the proposed conference is the Government's answer to the criticism which that action has called forth. We hope a satisfactory solution may speedily be reached, for London has waited too long for a modern system of street ambulances of the best kind.

Dr. Fernet has made an onslaught on tea and coffee in the *Semaine Medicale*. Dr. Fernet condemns both root and branch. According to him they have neither virtue nor merit, but are often to the habitual user as noxious drugs. A careful perusal of the facts set forth by Dr. Fernet tends, however, to mitigate, if it does not even destroy, most of his criticisms. It is undoubtedly the fact that China tea when properly made and poured off from the leaves into a hot pot may be kept hot and drunk with advantage by some brain workers for hours afterwards. The habitual use of coffee, where it is taken continuously throughout the day at most meals no doubt has a tendency to upset the digestion and to diminish health in many cases. Still coffee is a most useful stimulant when used in moderation by intelligent people who eat to live, as distinguished from that large number, who, nowadays, would seem to live to eat.

The services rendered to humanity by the work and research done in connection with the London and Liverpool Schools of Tropical Medicine have proved more momentous than almost anything which has happened in the last fifty years. One result has been to impress upon the directors of mining enterprises in unhealthy climates the importance of placing the hygienic and food arrangements for their staff of workers under the direction of a trained practitioner of medicine. The Gold Coast and the districts immediately surrounding it have been notorious for the ravages the climate and conditions have made upon European residents. Stricter attention to hygiene, the introduction of trained medical officers of health, and the enforcement of improved sanitation have produced remarkable results. These can be best expressed by the statement that the mortality amongst European workers in several portions of these countries has been diminished by 100 per cent. in the last few years.

This country certainly needs a powerful preacher of the gospel of recreation. What a terrible waste of human existence is the course pursued by the many and ever increasing thousands of people of both sexes in this country who for months together do nothing of a useful or intellectual character, but devote themselves entirely to games. We showed in a recent issue that upwards of £50,000 a year is paid to professional footballers in England today. What is the actual outlay entailed by the expenditure of those who devote all their energies mainly to such games as lawn tennis, golf, hockey, and even croquet? Relaxation implies the moderate use of amusement as a foil to and escape from the higher duties of life. In practice an ever increasing number of people are making games the serious purpose of their lives. They pursue them with the hope of gaining prizes for which money seems to be always forthcoming, and we have seen instances of young women who have crippled themselves or broken up their health by excessive indulgence in violent exercises. Hence the need of a preacher and of the enforcement of the gospel of relaxation.

